# The Human Figure

**Published:** 1891-05

**Authors:** 


### THE HUMAN FIGURE.

The rules of measurement used by Greek sculptors, are as follows :

From the crown to the nape of the neck is one-twelfth the stature of a perfectly formed man.

The hand from the wrist to the end of the middle finger is one-tenth of the total height of a man of perfect proportions.

A man of good proportions is as tall as the distance between the tips of his fingers when both arms are extended to full length.

The face from the highest point of the forehead, where the hair begins, to the end of the chin is one-tenth of the whole stature of a man of perfect mould.

If the face from the roots of the hair to the chin be divided into three equal parts, the first division determines the place where the eyebrows should meet, the second the opening of the nostrils, if the man be perfect in form.

The proportions of the human figure are six times the length of the right foot. Whether the form is slender or plump the rule holds good on an average. Any deviation from the rule is a departure from the beauty of proportion. It is claimed that the Greeks made all their statues according to this rule.

